# Supplementary material for: Computationally Reconstructed Interactome of Bradyrhizobium diazoefficiens USDA110 Reveals Novel Functional Modules and Protein Hubs for Symbiotic Nitrogen Fixation
Source: Int J Mol Sci. 2021 Nov 2;22(21):11907. doi: 10.3390/ijms222111907 (PMC8584416; doi:10.3390/ijms222111907)
Supplement: Supplementary file 1 [file ijms-22-11907-s001.zip › Supplementary information.pdf]

Computationally Reconstructed Interactome of *Bradyrhizobium diazoefficiens* USDA110 Reveals Novel Functional Modules and Protein Hubs for Symbiotic Nitrogen Fixation

Jun-Xiao Ma[#], Yi Yang[#], Guang Li and Bin-Guang Ma*

Hubei Key Laboratory of Agricultural Bioinformatics, College of Informatics, State Key Laboratory of Agricultural Microbiology, Huazhong Agricultural University, Wuhan 430070, China.

[#] These authors contribute equally to this work.
* Address correspondence to Bin-Guang Ma, mbg@mail.hzau.edu.cn

Supplementary Contents:

Supplementary Figures S1-S3.

Supplementary Table S1-S7.

Supplementary file.xlsx

Supplementary information

Supplementary figures

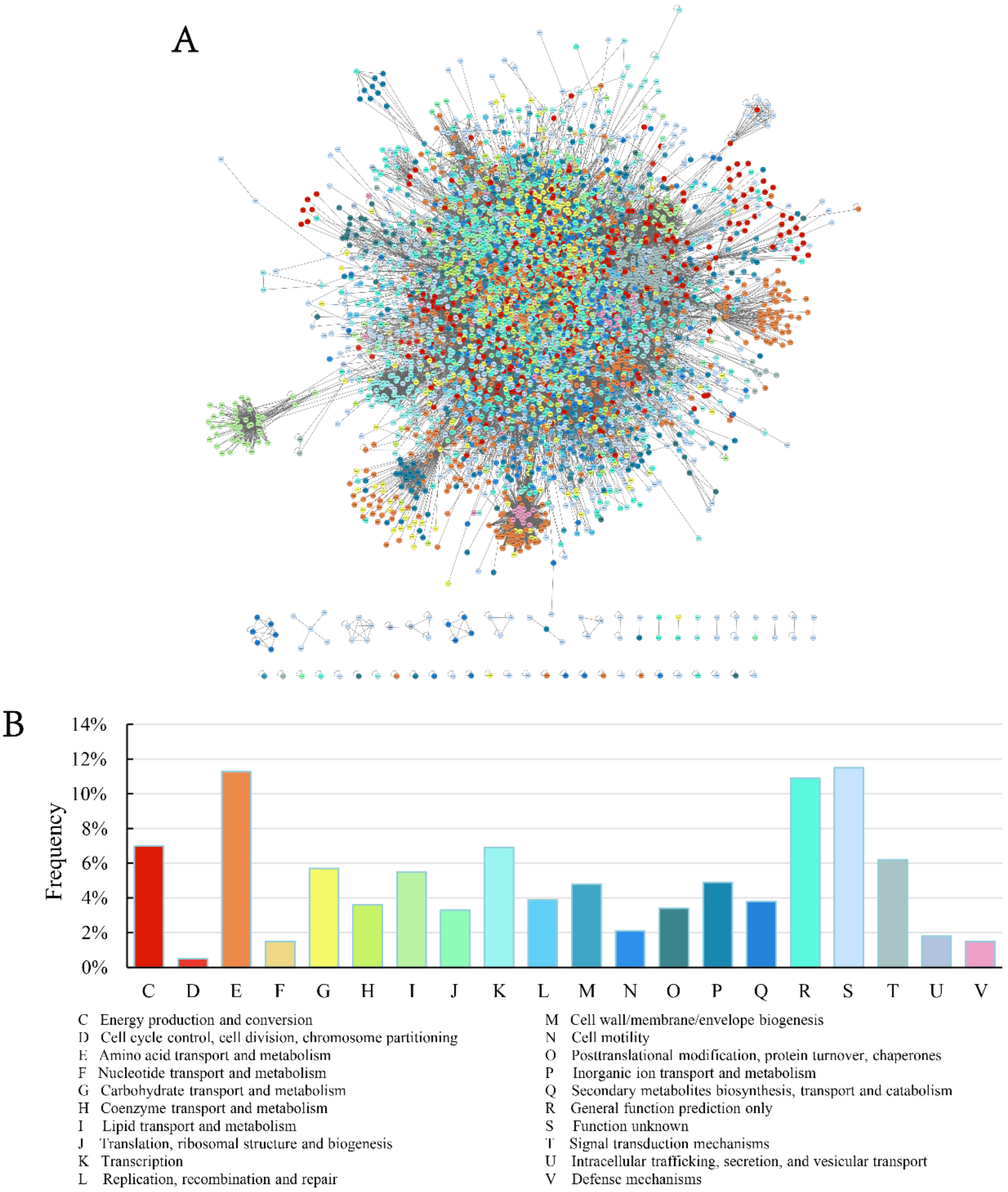

Figure S1. (A) The reconstructed protein-protein interaction (PPI) network of *B. diazoefficiens* USDA110. (B) The proportions of COG functional categories in the PPI network. The nodes in the PPI network were colored in accordance with COG functional categories.

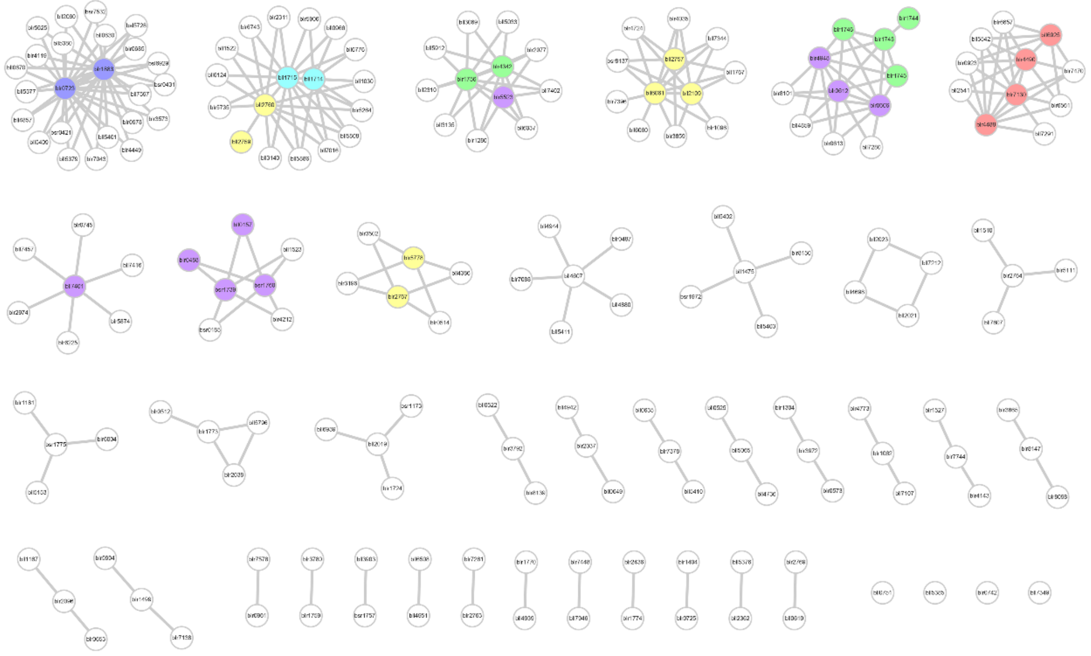

Figure S2. Functional modules of SNF core-sub-network in the PPI network of *B. diazoefficiens* USDA110 identified by MCL clustering. The modules were arranged in descending order by module size and the first 9 modules (m1-m9) were colored. The node colors represent different kinds of SNF-associated proteins: purple, sigma54 factor; cyan, nod proteins; yellow, fix proteins; green, nif proteins; red, two component regulators; violet, other SNF-associated proteins.

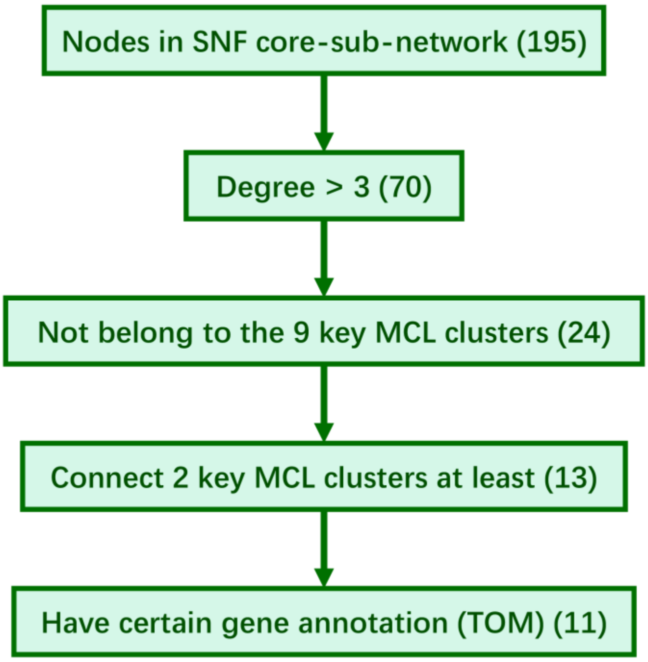

Figure S3. Schematic representation of the procedure used to select hubs with specific properties named Tie of Modules (TOM) in the SNF core-sub-network of *B. diazoefficiens* USDA110 PPI network.

Supplementary tables

Table S1. Significantly enriched COG categories in the MDS of the reconstructed PPI network (fisher's exact test, *p*-value<0.01).

COG category	Number of proteins in PPI network	Number of proteins in MDS	*p*-value
V Defense mechanisms	96	6	6.53e-6
I Lipid transport and metabolism	349	8	1.49 e-4
O Posttranslational modification, protein turnover, chaperones	210	25	2.01e-3
U Intracellular trafficking, secretion, and vesicular transport	61	11	7.78 e-3
C Energy production and conversion	414	37	9.72 e-3

Table S2. Conserved functional modules of the global PPI networks of *B. diazoefficiens* USDA110 and *M. loti* obtained by the GASOLINE algorithm.

Module	*B. diazoefficiens* USDA110	*M. loti*	ISC score	Module size
1	blr3189,bll7021,blr7895,blr3104	Q98IV9,Q98BL5,Q98DQ2,Q98M07	1.00	4
2	bll1799,bll8025,bll1317,bll3903	Q989N3,Q98ID9,Q98AS5,Q98B05	1.00	4
3	blr4439,bll2403,bll5459,bll7190	Q98K28,Q98H78,Q98K67,Q987J9	1.00	4
4	bll4303,bll0933,blr1822,bll3903	Q98GT2,Q983Q0,Q989N6,Q98B05	1.00	4
5	blr7523,bll7180,bll2795,blr4388,bll2590	Q987U8,Q98DA7,Q98P62,Q98JF1,Q988U1	0.96	5
6	blr3074,bll6508,bll3903,blr4773,blr6751	Q985J1,Q98KI9,Q98B05,Q98LK8,Q98IT8	0.96	5
7	bll6164,bll6258,bll8025,bll3903,blr3064	Q98CI8,Q983P9,Q98ID9,Q98B05,Q98GJ6	0.96	5
8	bll5925,blr0616,blr8186,bll3903,bsl1473	Q985Q9,Q98EU9,Q98KE1,Q98B05,Q98PA0	0.96	5
9	bll3903,bll5925,blr2027,blr6216	Q98B05,Q985Q9,P17862,Q98LU4	0.94	4
10	bll7306,bll2759,bll2760,bsl5082	Q98DH9,Q988T4,Q988T3,Q988T5	0.94	4
11	blr7730,blr2168,blr4157,bll3551	Q983H9,Q98B19,Q98ME0,Q986X5	0.94	4
12	bll3246,bll6292,bsr6998,bll6711	Q98FP1,Q98MG5,Q98HL2,Q987J3	0.94	4
13	blr2027,blr6216,bll5925,bll3903	P17862,Q98LU4,Q985Q9,Q98B05	0.94	4
14	blr0425,bll6773,bll3903,bll1317	Q98EZ3,Q98P39,Q98B05,Q98AS5	0.94	4
15	bll0465,blr0616,bll0109,blr8186	Q98EA1,Q98EU9,Q98L85,Q98KE1	0.94	4
16	bll8024,bll0109,bll5913,bll3903,blr6172	Q98B49,Q98L85,Q985K2,Q98B05,Q98GX4	0.92	5
17	blr5104,blr5132,bll1799,blr7359,bll4958	Q98M83,Q98MA8,Q989N3,Q98E49,Q989W9	0.92	5
18	bll0378,blr5523,bll5913,bll3903,blr6753	Q986V5,Q98LQ7,Q985K2,Q98B05,Q98IU1	0.92	5
19	bll2590,bll2795,bll0109,blr4388,bsl0578	Q988U1,Q98P62,Q98L85,Q98JF1,Q98ET2	0.92	5
20	bll0457,bll6508,bll4898,bll0109,blr6172	Q98EC2,Q98KI9,Q986M8,Q98L85,Q98GX4	0.92	5
21	blr1228,blr4388,blr0711,bll0109,blr3064	Q98CW4,Q98JF1,Q98CP4,Q98L85,Q98GJ6	0.92	5
22	bll1475,bll0109,blr3873,bll5663,blr1095	Q985Q4,Q98L85,Q98K79,Q98NL5,Q98BC0	0.88	5
23	bll0109,blr6774,bll4898,bll5663,bll1475	Q98L85,Q98DP7,Q986M8,Q98NL5,Q985Q4	0.84	5

24	blr0329,bll0659,bll3140,blr3467	Q98L88,Q98FG5,Q985F5,Q98L87	0.81	4
25	blr1194,bll2599,bll1199,blr7015,blr2192,blr2194	Q98B86,Q98FG5,Q98L88,Q98L87,Q98PD0,Q98HL7	0.81	6
26	bll4573,blr2192,bll0659,blr6651,blr2194,bll3706	Q98HI5,Q98PD0,Q98FG5,Q98E43,Q98HL7,Q98B86	0.78	6

Table S3. Conserved functional modules of the PPI networks of *B. diazoefficiens* USDA110 and *S. meliloti* under SNF state obtained by the GASOLINE algorithm.

Module	*B. diazoefficiens* USDA110	*S. meliloti*	ISC score	Module size
1	blr1170,blr0150,blr1175,blr0151	Q92RH0,Q92U26,Q92RG5,Q92Z14	0.88	4
2	bll6899,blr3345,blr5675,bll4543	Q92RF4,Q92MM9,Q92MN0,Q92MM8	0.75	4
3	blr5675,bll6899,bll4542,bll4543	Q926C5,Q92MN0,Q92MX4,Q92MX3	0.75	4
4	blr3725,bll3808,blr2146,blr0770	P50205,P06230,P06234,Q92SV9	0.69	4
5	blr2146,bll3808,blr0770,blr3725	P06234,Q92P54,P06230,P50205	0.69	4
6	blr4490,blr4488,blr7130,bll7291	Q92Q87,P13632,P10577,P03028	0.69	4
7	blr4488,blr5735,blr5264,bll5808,bll0966,blr3071	P10577,P03028,Q92Q87,Q52977,P13632,Q92TC2	0.67	6
8	bll3809,blr3725,bll3808,blr0770	P56902,P06234,P06230,Q92SV9	0.63	4
9	blr6078,blr5675,bll7146,bll6899	Q92MN0,Q92ZZ6,Q92MM9,Q926C5	0.63	4
10	blr3353,bll7146,bll6899,blr5675	Q92ZZ6,Q92MM9,Q92MN0,Q926C5	0.63	4

Table S4. Functional annotation of the proteins in COG category S ("function unknown") by using the conserved PPI modules in *B. diazoefficiens* USDA110 and *M. loti* (network comparison was performed by using the GASOLINE algorithm; functions are inferred based on interacting partners and GO and KEGG annotations; The highlighted genes have no annotations in GO and KEGG databases and purely predicted by the PPI networks).

B. diazoefficiens USDA110	COG	Annotation of gene function	*M. loti*	COG	Annotation of gene function
bll8024	S	ATP binding	Q98B49	S	ATP binding
blr6172	S	cellular response to DNA damage stimulus	Q98GX4	S	cellular response to DNA damage stimulus
blr6753	S	transcriptional regulator related to branched-chain amino acid biosynthesis	Q98IU1	S	transcriptional regulator related to branched-chain amino acid biosynthesis
blr6774	S	double-stranded DNA binding	Q98DP7	S	double-strand break repair via nonhomologous end joining
blr3189	S	integral component of membrane	Q98IV9	S	integral component of membrane
blr7895	S	metal ion binding；oxidoreductase activity	Q98DQ2	S	metal ion binding；oxidoreductase activity

blr6216	S	formaldehyde catabolic process	Q98LU4	S	formaldehyde catabolic process
bll0465	S	peptidase related to abruption of rhizobia	Q98EA1	S	peptidase related to abruption of rhizobia
blr8186	S	transferase activity	Q98KE1	S	transferase activity
blr7523	S	16S ribosomal RNA methyltransferase RsmE	Q987U8	S	16S ribosomal RNA methyltransferase RsmE
blr3074	-	the process of coordination between nitrogen fixation, nodulation and nitrogen assimilation	Q985J1	-	the process of coordination between nitrogen fixation, nodulation and nitrogen assimilation
blr6751	S	response to stress	Q98IT8	S	response to stress
blr8186	S	transferase activity	Q98KE1	S	transferase activity
bsl1473	S	transcriptional regulator related to abruption of rhizobia	Q98PA0	S	transcriptional regulator related to abruption of rhizobia
bsl0578	S	peptidase related to amino acid metabolism	Q98ET2	S	peptidase related to amino acid metabolism
blr6172	S	cellular response to DNA damage stimulus	Q98GX4	S	cellular response to DNA damage stimulus

Table S5. Comparison of normalized transcription level differences of the interacting proteins belonging to the same COG category under FL and SNF states (the groups with significant differences are shown, one-sided Wilcoxon rank sum test, *α* = 0.05).

COG category	No. of PPIs under FL state	No. of PPIs under SNF state	D_{ij} Median under FL state	D_{ij} Median under SNF state	*p*-value
Energy production and conversion (C)	638	349	0.26	0.82	<2.20e-16
Carbohydrate transport and metabolism (G)	490	116	0.35	0.14	6.48e-8
Translation, ribosomal structure and biogenesis (J)	421	166	0.82	0.81	2.85e-3
Cell wall/membrane/ envelope biogenesis (M)	329	89	0.82	0.82	0.0152
Secondary metabolites biosynthesis, transport and catabolism (Q)	519	239	0.82	0.77	1.29e-6

Table S6. The 128 SNF-associated proteins in *B. diazoefficiens* USDA110 determined by genome annotation and literature mining.

SNF-associated proteins in *B. diazoefficiens* USDA110								
bll0157	bll0416	blr0488	blr0517	blr0606	blr0612	blr0653	blr0680	blr0723
blr0724	blr0725	blr0742	bll0800	blr0906	blr1062	blr1063	bll1167	blr1383
bll1421	bll1475	blr1499	bll1630	bll1631	blr1632	bll1714	bll1715	bsr1739
blr1743	blr1744	blr1745	blr1746	blr1755	blr1756	bsr1757	blr1759	bsr1760

**blr1769**	**blr1773**	**blr1774**	**bsr1775**	**blr1813**	**blr1815**	**blr1883**	**blr1902**	**bll2019**
**bll2021**	**bll2023**	**blr2026**	**blr2027**	**blr2028**	**blr2029**	**blr2030**	**blr2031**	**blr2033**
**blr2034**	**blr2036**	**blr2037**	**blr2038**	**blr2062**	**bll2109**	**bll2362**	**blr2369**	**bll2623**
**blr2694**	**bll2757**	**bll2759**	**bll2760**	**blr2763**	**blr2764**	**blr2766**	**blr2767**	**blr2769**
**blr2807**	**blr3125**	**blr3126**	**blr3127**	**blr3128**	**bll3460**	**bll3466**	**blr3792**	**blr3959**
**blr3972**	**blr4338**	**blr4342**	**blr4486**	**blr4487**	**blr4488**	**blr4489**	**blr4490**	**bll4607**
**bll4612**	**blr4614**	**blr4948**	**bll4951**	**bll4952**	**bll5061**	**bll5065**	**bll5464**	**bll5465**
**bll5476**	**blr5523**	**blr5778**	**bll6061**	**bll6925**	**blr7003**	**blr7130**	**bll7191**	**bll7193**
**blr7378**	**bll7401**	**blr7496**	**blr7573**	**bll7574**	**blr7577**	**blr7578**	**bll7696**	**blr7744**
**bll7861**	**blr7884**	**blr7900**	**bll7944**	**bll7945**	**bll7946**	**bll7947**	**blr8147**	**blr2768**
**blr1770**	**blr1771**							

Table S7. Deduced functions of TOMs (Tie of Modules).

Synonym	Gene	COG	SNF associated proteins or not	Degree	Connected modules and TOMs	Annotation of gene function	Deduced function of related PPIs	Refs
bll7349	sigA	K	N	11	m2 m4 m5 m6 blr8147	RNA polymerase sigma factor RpoD	Regulating binding strength of sigma factor RpoD to its binding sites, further affects (mainly reduces) the transcription of certain housekeeping genes (especially carbon metabolism-related) that are actively transcribed in FL state	-
blr4773	nwsA	T	N	10	m2 m4 m6 blr1499 blr2037	Two-component hybrid sensor and regulator	Coordination between nitrogen fixation, nodulation and nitrogen assimilation.	[1-8]
blr0742	ihfB	L	Y	20	m1 m7 m8 blr3780	Integration host factor subunit beta	Influencing (mainly enhancing) gene expression	[9]
blr3780	nadE	H	N	8	m3 m6 blr1499 blr0742	NAD synthetase	-	-
blr1499	exoN	M	Y	11	m2 m7 blr4773 blr3780	UTP-glucose-1-phosphate uridylyltransferase	-	-
bll4944	clpP	OU	N	5	m1 m3 m5	ATP-dependent Clp protease proteolytic subunit	Hydrolysis of abnormal SNF associated proteins	[10]
blr0725	ptsN	GT	Y	8	m2 m4 m6	Nitrogen regulatory IIA protein	-	-
bll5385	rplF	J	N	6	m3 m7 m8	50S ribosomal protein L6	-	-
blr8147	-	G	Y	7	m1 m2 bll7349	PTS system transporter subunit IIA	-	-
blr2037	nifA	KT	Y	8	m2 m6 blr4773	Nif-specific regulatory protein	Coordination between nitrogen fixation, nodulation and nitrogen assimilation.	[1-8]
blr7448	prsA	FE	N	5	m4 m8	Ribose-phosphate pyrophosphokinase	-	-

1. Grob, P. et al. A novel response-regulator is able to suppress the nodulation defect of a Bradyrhizobium japonicum nodW mutant. *Mol Gen Gene.* 1993, 241: 531-541
2. Loh, J and Stacey G. Nodulation Gene Regulation in Bradyrhizobium japonicum: a Unique Integration of Global Regulatory Circuits. *Appl Environ Microb*. 2003, 69: 10-17
3. Nellen-Anthamatten, D. et al. Bradyrhizobium japonicum FixK2, a crucial distributor in

the FixLJ-dependent regulatory cascade for control of genes inducible by low oxygen levels. *J. Bacteriol*. 1998, 180: 5251–5255.
4. Dixon R. and D. Kahn. Genetic regulation of biological nitrogen fixation. *Nat. Rev. Microbiol.* 2004. 2: 621–631
5. Mesa S. et al. Comprehensive Assessment of the Regulons Controlled by the FixLJ-FixK2-FixK1 Cascade in Bradyrhizobium japonicum. *J. Bacteriol*. 2008, 190: 6568–6579
6. Lindemann A, Moser A, Pessi G, Hauser F, Friberg M, Hennecke H, Fischer HM. New target genes controlled by the Bradyrhizobium japonicum two-component regulatory system RegSR. *J Bacteriol*. 2007, 189: 8928-8943
7. Martin G. B. et al. Role of the Bradyrhizobium japonicum ntrC Gene Product in Differential Regulation of the Glutamine Synthetase II Gene (glnII). *J. Bacteriol*. 1988, 170: 5452-5459
8. Franck W. L. et al. DNA Microarray-Based Identification of Genes Regulated by NtrC in Bradyrhizobium japonicum. *Appl Environ Microb*. 2015, 81: 5299 –5308.
9. Stonehouse E. et al. Integration Host Factor Positively Regulates Virulence Gene Expression in Vibrio cholerae. *J. Bacteriol*. 2008, 190: 4736–4748
10. Wang J. et al. The Structure of ClpP at 2.3 A Resolution Suggests a Model for ATP-Dependent Proteolysis. *Cell*. 1997, 91: 447–456

Supplementary file.xlsx

The reconstructed protein-protein interaction networks of *Bradyrhizobium diazoefficiens* USDA110 in different situations.
